# MARCKS Is an Essential Regulator of Reactive Oxygen Species Production in the Monocytic Cell Type

**DOI:** 10.3390/antiox11081600

**Published:** 2022-08-18

**Authors:** René Huber, Mareike Diekmann, Leonie Hoffmeister, Friederike Kühl, Bastian Welz, Korbinian Brand

**Affiliations:** Institute of Clinical Chemistry, Hannover Medical School, 30625 Hannover, Germany

**Keywords:** monocytes, ROS, MARCKS, PKCβ, wildtype, knockout, Akt, TNF

## Abstract

Myristoylated alanine-rich C-kinase substrate (MARCKS) is a ubiquitous protein mediating versatile effects in a variety of cell types, including actin crosslinking, signal transduction, and intracellular transport processes. MARCKS’s functional role in monocyte/macrophages, however, has not yet been adequately addressed. Thus, the aim of this study was to further elucidate the impact of MARCKS on central cellular functions of monocytic cells. To address this topic, we generated monocytic THP-1 (Tohoku Hospital Pediatrics-1)-derived MARCKS wildtype and knockout (KO) cells using the CRISPR/Cas9 technique. Remarkably, in the absence of MARCKS, both total and intracellular reactive oxygen species (ROS) production were strongly suppressed but restored following transient MARCKS re-transfection. In contrast, proliferation, differentiation, cytokine expression, and phagocytosis remained unaltered. A complete inhibition of ROS production could also be achieved in THP-1-derived PKCβ KO cells or in PKC inhibitor Staurosporine-treated primary human monocytes. MARCKS deficiency also involved reduced basal Akt phosphorylation and delayed re-phosphorylation. Further analyses indicated that long-term TNF pre-incubation strongly enhances monocytic ROS production, which was completely blocked in MARCKS and PKCβ KO cells. Collectively, our study demonstrates that MARCKS is an essential molecule enabling ROS production by monocytic cells and suggests that MARCKS is part of a signal cascade involved in ROS formation.

## 1. Introduction

Myristoylated alanine-rich C-kinase substrate (MARCKS) is a ubiquitous alanine-rich, unstructured, acidic 32 kDa protein characterized by a myristoylated N-terminal domain, a MARCKS homology 2 (MH2) domain of unknown function, and a lysine-rich effector domain (ED) consisting of a phosphorylation site with four serine residues [1,2]. In the unphosphorylated state, the protein associates with the cell membrane [3] via its basic ED and the N-terminal myristoyl group [4]. At the membrane, MARCKS mediates a number of effects, including the crosslinking of filamentous actin [5], the sequestration of phosphatidylinositol 4,5-bisphosphate (PIP_2_) in lipid rafts within the membrane [6], and the binding of vesicle traffic-regulating proteins such as Ras-related GTP-binding protein 10 (Rab10) [7], thus supporting exocytosis [2]. Following ED phosphorylation by kinases, such as protein kinase C (PKC) and Rho-associated coiled-coil containing kinase (ROCK), or the binding of calmodulin to the ED in the presence of high Ca^2+^ levels, MARCKS translocates to the cytosol [5,8]. By dissociating from the membrane, MARCKS enables the activation of PIP_2_-dependent signaling pathways, e.g., the PI3K/Akt (via PIP_3_) [9] or the PLC/PKC axis (via diacylglycerol) [6].

In consequence, MARCKS is involved in the regulation of a variety of cellular processes such as cytoskeletal reorganization, mitosis, adhesion, migration, vesicle trafficking, secretion, and signal transduction [10]. Accordingly, disturbances in MARCKS level or function are associated with numerous pathophysiological conditions [11], e.g., pulmonary [12] or neuronal diseases [13], cancer and metastasis [1,14], or fibrosis [15]. Since these cellular functions are also of decisive importance in immune cells, MARCKS plays a relevant role in the immune response [11]. For instance, MARCKS has been reported to be involved in the regulation of B cell receptor signaling [16], macrophage [17,18] and neutrophil migration and adhesion [19,20], macrophage phagosome maturation [21], and neutrophil cytokine expression [22]. In monocytic cells, MARCKS plays a role in degranulation [23] and protein secretion [24] as well as in the expression of pro-inflammatory cytokines, including tumor necrosis factor (TNF) and interleukin 6 (IL-6) [25]. Moreover, in long-term TNF-incubated primary human monocytes, a significant increase in MARCKS phosphorylation in the linker region connecting MH2 and ED was observed [26]. MARCKS’s global functional role in monocyte/macrophages, however, has not yet been adequately addressed. Thus, the aim of this study was to further elucidate the impact of MARCKS on central cellular functions of monocytic cells. To address this topic, monocytic THP-1-derived MARCKS wildtype (WT) and knockout (KO) cells were generated using the CRISPR/Cas9 technique [27,28], and several cellular characteristics defining the monocyte’s immunologic functions were analyzed. The present study demonstrates that MARCKS plays a crucial role in monocytic ROS production.

## 2. Materials and Methods

### 2.1. CRISPR/Cas9-Mediated Generation of WT, Intermediate (IM), and KO Cells

#### 2.1.1. CRISPR/Cas9 Plasmids

The MARCKS- and PKCβ-targeting CRISPR/Cas9 plasmids PX458-MARCKS and PX458-PKCβ used in this study were based on the Cas9- and green fluorescent protein (GFP)-expressing plasmid pSpCas9(BB)-2A-GFP (PX458; plasmid #48138, Addgene, Watertown, MA, USA) generated by Feng Zhang and processed according to [29] using suitable target sequences (see below).

#### 2.1.2. Transfection of THP-1 Cells with CRISPR/Cas9 Plasmids

Before nucleofection, THP-1 human acute monocytic leukemia cells [30] were cultured for two passages in advanced culture medium (9% FCS, 0.9 mM Hepes (Biochrom, Berlin, Germany), 0.9 mM pyruvate, 0.9× non-essential amino acids, 5.2 mM glutamine, 40 U/mL penicillin, and 40 μg/mL streptomycin). Nucleofection was performed using the Nucleofector Kit T (Lonza, Basel, Switzerland) according to the manufacturer’s protocol. A total of 2 × 10^6^ cells were suspended in 98 μL T-solution, and 5 μg of the CRISPR/Cas9 plasmids was added. Cells were transfected with a Nucleofector II (program T-020; Lonza). Transfected cells were transferred into a 6-well plate containing advanced culture medium. One day post-nucleofection, EDTA was added (final concentration: 5 mM). Single cell clones were obtained by sorting GFP-positive cells into 96-well plates (Corning, Corning, NY, USA) containing 200 μL advanced culture medium using a FACSAria Fusion (BD Bioscience, Heidelberg, Germany) at the MHH central research facility cell sorting. Cell clones were expanded and stored at −80 °C for further use (1 × 10^6^ cells/mL in FCS containing 10% DMSO).

#### 2.1.3. Generation of MARCKS WT, IM, and KO Clones

Since THP-1 cells contain 3 MARCKS alleles due to leukemia-associated genetic alterations [31], we aimed at developing a strategy to create both a complete KO and an intermediate (IM) genotype (possessing either 1 or 2 MARCKS alleles). Therefore, the CRISPR/Cas9 technique was applied [27,28] using gRNAs derived from the Brunello library [32]. The following sequences were targeted: 5′-AAGAAGTCTTTCAAGCTGAG-3′ (#1), 5′-CTCACCTTTCTCGGCCGCGG-3′ (#2), and 5′-TCGTCGCCTTCCAAAGCGAA-3′ (#3). THP-1 cells were transfected with the CRISPR/Cas9 plasmids (see above), and the respective clones were analyzed for MARCKS protein expression using Western Blot and flow cytometry (see below). The integrity of the MARCKS alleles was assessed via cycle sequencing (see below), and MARCKS WT (3 intact alleles), MARCKS IM (2 intact alleles), and MARCKS KO clones (0 alleles) were identified.

#### 2.1.4. Generation of PKCβ KO Clones

For the generation of PKCβ KO clones, the following target sequences were selected: 5′-CCACAGTGGTCACAAAACGT-3′ (#1), 5′-CTTGCTGGATGTGATACATG-3′ (#2), and 5′-TGACGTGGAGTGCACTATGG-3′ (#3).

#### 2.1.5. Western Blot

MARCKS protein levels in whole cell extracts of WT, IM, and KO cells as well as PKCβ levels in PKCβ KO cells were initially detected using the Western Blot technique as previously described [33]. For protein detection, membranes were incubated (4 °C, overnight) with primary antibodies specific for MARCKS (D88D11 XP^®^), phosphorylated (p)-MARCKS (Ser167/170; D13E4 XP^®^), PKCβ (D3E70), Akt (C67E7), or p-Akt (Thr308; D25E6 XP^®^; Cell Signaling Technology, Danvers, MA, USA) and glyceraldehyde-3-phosphate dehydrogenase (GAPDH; Sigma Aldrich, St. Louis, MO, USA). Following incubation with horseradish peroxidase (HRP)-coupled secondary antibodies (Vector Laboratories/Alexis, Grünberg, Germany), protein bands were visualized using the detection reagents enhanced chemiluminescence (ECL; Thermo Fisher, Bonn, Germany) or WesternBright Sirius (Advansta, Menlo Park, CA, USA) and the Bio-imaging system ECL Chemostar (Intas Science Imaging, Göttingen, Germany).

#### 2.1.6. Flow Cytometry

For the flow cytometry-based detection of MARCKS expression levels, an intracellular staining approach was used. Fixed and permeabilized cells were incubated with the MARCKS (D88D11 XP^®^) antibody (Cell Signaling) for 1 h and subsequently labeled with the Alexa Fluor 647 AffiniPure F(ab’)₂ Fragment (Jackson ImmunoResearch, West Grove, PA, USA) for 1 h. For detection, a FACSCanto II flow cytometer and the FACSDiVa software (BD Biosciences) were applied. The obtained data were further processed using FlowJo (BD Bioscience) and GraphPad PRISM 5.02 (GraphPad Software, La Jolla, CA, USA).

#### 2.1.7. Cycle Sequencing

To assess the integrity of MARCKS and PKCβ alleles following the CRISPR/Cas9 procedure, genomic DNA from MARCKS WT, IM, and KO as well as PKCβ KO clones was isolated using the QIAamp DNA Mini Kit (Qiagen, Hilden, Germany) according to manufacturer’s instructions. Concentration and purity were assessed using the Nanodrop ND-1000 (PeqLab Biotechnologie, Erlangen, Germany). The targeted DNA sequences were amplified (primers: MARCKS KO approaches #1 and #2: 5′-AGCTGCAGGCCAACGGCAGCGC-3′, 5′-TGCGCCCCCGGCGGCCTCGT-3′, MARCKS KO #3: 5′-TGTTTCCCCTCTTGGATCTGT-3′, 5′-TCCACGAATGAGCCTTGGGA-3′; PKCβ KO approach #1: 5′-TGCAAAGGGAACAATCATCT-3′, 5′-ACTTGCCCTCCTAAGAAACT-3′, #2: 5′-TGTGTATCACCTATGCTCACT-3′, 5′-TAGGGCCTGGCTTATAGTAAGT-3′, and #3: 5′-TGCTATTGCAATCTAGGCTGGGT-3′, 5′-TCCAATGAGGCCCATGCAAA-3′) and cloned into the vector pCR-Blunt II-TOPO using the Zero Blunt TOPO PCR cloning kit (Invitrogen, Darmstadt, Germany). Subsequently, the genetic condition of MARCKS and PKCβ alleles was confirmed by commercial cycle sequencing (Eurofins Genomics, Ebersberg, Germany). Resulting sequencing data were analyzed and processed using Chromas (Technelysium, South Brisbane, Australia), CLC Sequence Viewer 8 (Qiagen), and SnapGene Viewer 4.1.1 (GSL Biotech, Chicago, IL, USA).

### 2.2. Isolation of Primary Human Monocytes and Cell Culture Conditions

#### 2.2.1. Isolation of Primary Monocytes

Freshly obtained blood samples from healthy donors were provided by the Institute of Transfusion Medicine, Hannover Medical School. Informed donor consent was obtained, and the experiments were approved by the Hannover Medical School ethics committee in accordance with the Declaration of Helsinki. Monocytes were isolated as previously described [33] using the Monocyte Isolation Kit II or the Pan Monocyte Isolation Kit (Miltenyi Biotec, Bergisch Gladbach, Germany) and incubated overnight before further use. The purity of isolated primary human monocytes was assessed by dual cell labeling for 30 min (4 °C, dark) using Alexa Fluor 405-CD45 (Invitrogen) and allophycocyanin-CD14 (BD Biosciences) recombinant human antibodies. Contamination with other leukocytes was excluded using the antibodies allophycocyanin-CD3, PE-CD19, FITC-CD56 (BD Biosciences), and FITC-CD15 (Invitrogen) [34].

#### 2.2.2. Cell Culture

Human monocytic THP-1 cells were purchased from the Deutsche Sammlung von Mikroorganismen und Zellkulturen (DSMZ, Braunschweig, Germany). Primary monocytes, THP-1, and THP-1-derived WT, IM, and KO cells were maintained in Roswell Park Memorial Institute 1640 medium including 100 U/mL penicillin, 100 mg/mL streptomycin (Biochrom), and 7.5% fetal calf serum (FCS; Sigma Aldrich). Medium for primary monocytes was additionally supplemented with 2% oxaloacetate/pyruvate/insulin media supplement (Sigma Aldrich) and 1% minimum essential medium non-essential amino acids solution (Thermo Fisher). THP-1 cells and primary human monocytes were cultured at a density of 5 × 10^5^ cells/well in 12-well plates (Sarstedt, Nümbrecht, Germany).

#### 2.2.3. Inhibitors and Activators

PKC inhibition was performed using Staurosporine (Stauro; Sigma Aldrich). For experiments including TNF preincubation, human TNF from Sigma Aldrich or Peprotech (Peprotech, Rocky Hill, CT, USA) was used. All media and reagents were of the best available grade and routinely tested for endotoxins with the Limulus Amoebocyte Lysate assay (Lonza).

#### 2.2.4. Differentiation of THP-1 and THP-1-Derived Cells

Differentiation of THP-1 and THP-1-derived cells was performed with 100 nM calcitriol (Peprotech) [35] and confirmed by flow cytometry using the Alexa Fluor 647-coupled CD14 antibody (Biolegend, San Diego, CA, USA). Though flow cytometry-based analysis of CD14 expression at day 3 confirmed the successful differentiation towards the monocytic phenotype, the cells were generally cultivated for 5 d in the presence of calcitriol to prevent de-differentiation in the experiments in which TNF was added for a further 48 h.

### 2.3. Detection of Total and Intracellular ROS Production

#### 2.3.1. ROS-Inducing Stimuli

For the induction of ROS production, either 100 nM phorbol 12-myristate 13-acetate (PMA; Sigma Aldrich) or opsonized Top10 *E. coli* bacteria (Invitrogen) were used. For opsonization, frozen *E. coli* were suspended in Hank’s balanced salt solution (HBSS) with magnesium and calcium (Thermo Fisher), centrifuged (11,000× *g*, 2 min), and incubated with 50% pooled human complement serum (Innovative Research, Novi, MI, USA) in HBSS for 30 min (shaking at 600 rpm). Afterwards, opsonized bacteria were washed and resuspended in HBSS to achieve a dilution of more than five bacteria per cell used in the subsequent assays.

#### 2.3.2. Luminometer-Based Analysis of ROS

In most cases, total and intracellular ROS were detected by chemiluminescence, i.e., the HRP-catalyzed conversion of luminol (5-amino-2,3-dihydro-1,4-phtalazinedione) in the presence of ROS (e.g., hydrogen peroxide, hydroxyl radicals, hypochlorous acid, or peroxynitrite). Therefore, 2–4 × 10^5^ cells were seeded in 40 μL HBSS in white Costar 96-well assay plates (Corning). After 30 min at 37 °C, 40 μL of 2× ROS detection solution (for total ROS: 0.1 mM luminol and 1 U/mL horseradish peroxidase in HBSS; for intracellular ROS: 0.1 mM luminol, 50 U/mL superoxide dismutase, and 2000 U/mL catalase in HBSS; Sigma Aldrich) was added. ROS production was induced using PMA (injected by the luminometer) or opsonized bacteria (added before starting the assay) and quantified as relative light units (RLU) using an Orion L microplate luminometer (Berthold Detection Systems, Pforzheim, Germany). Data were analyzed and processed using GraphPad PRISM 5.02.

#### 2.3.3. Flow Cytometry-Based Analysis of ROS

For the analysis of intracellular ROS production in re-transfected MARCKS KO cells (see below), a flow cytometry-based approach using CellROX DeepRed Reagent (Thermo Fisher) was applied. CellROX DeepRed is a cell-permeable dye specifically indicating the presence of ROS via fluorescence emission following ROS-dependent oxidation. Therefore, 5 × 10^4^ cells were seeded in 50 μL medium in a 96-well v-bottom plate (Corning) and stimulated with 100 nM PMA for 30 min. Afterwards, the cells were harvested, washed with PBS, and incubated in 100 μL medium containing 5 μM CellROX Deep Red Reagent for 30 min at 37 °C. Subsequently, cells were washed twice with PBS (4 °C), suspended in FACS buffer, mingled with 4′,6-diamidino-2-phenylindole (DAPI; Biolegend), and analyzed using the FACSCanto II. Data were analyzed using FlowJo and GraphPad PRISM 5.02.

### 2.4. MARCKS Reconstitution in MARCKS KO Cells

#### 2.4.1. MARCKS Expression Plasmid

The reconstitution of MARCKS expression in MARCKS KO cells was performed using the expression plasmid pcDNA 3.1 V5H6-A MARCKS-WT consisting of the full length human MARCKS coding sequence (amplified using the primers 5′-TCGAATTCATGGGTGCCCAGTTCT-3′ and 5′-TGGATCCTCCCTCTGCCGCCTCC-GCT-3′) cloned into the vector pcDNA 3.1/V5-His A (Invitrogen) via the restriction sites EcoRI and EcoRV.

#### 2.4.2. Transfection of MARCKS KO Cells

A total of 5 × 10^5^ three-day calcitriol-differentiated MARCKS KO cells/well in 500 μL medium (incl. 10% FCS) were transfected using X-tremeGENE HP DNA Transfection Reagent (Roche, Rotkreuz, Switzerland) according to manufacturers’ instructions. In addition, 400 ng pcDNA 3.1 V5H6-A MARCKS-WT, 100 ng pEGFP N3 control plasmid (Clontech, Mountain View, CA, USA ; for monitoring transfection efficacy), and transfection reagent (volume ratio reagent to nucleic acid: 3:1) were preincubated in 100 μL Opti-MEM I (Thermo Fisher) for 30 min and then added to the cells. Six hours post-transfection, the medium was replaced to remove the remaining transfection mixture, and cells were incubated in the presence of calcitriol until day 5. Then, MARCKS expression levels were determined by Western Blot, and intracellular ROS production was assessed by flow cytometry.

### 2.5. Proliferation Assay

The proliferation of MARCKS WT and KO cells was analyzed using 1.25 × 10^5^ cells synchronized in starvation medium (1% FCS) for 3 d. Subsequently, cells were transferred into standard medium at a density of 5 × 10^4^ cells/mL. For 5 d, cells were counted daily in sextuplicates using a Neubauer chamber (W. Schreck, Hofheim, Germany).

### 2.6. Determination of Cytokine Expression

The cytokine expression in monocytic cells was assessed using quantitative polymerase chain reaction (qPCR). Cell lysis, total RNA isolation, reverse transcription of total RNA [36], and qPCR [33] were performed as previously described. The following primers were applied: interleukin (IL-)1α (5′-TGACTGCCCAAGATGAAGAC-3′, 5′-CCAAGCACACCCAGTAGTC-3′), IL-1β (5′-CTCGCCAGTGAAATGATGGCT-3′, 5′-GTCGGAGATTCGTAGCTGGAT-3′) [37], IL-6 (5′-ACAGCCACTCACCTCTTCAG-3′, 5′-GTGCCTCTTTGCTGCTTTCAC-3′), CC motif chemokine ligand (CCL)20 (5′-GAAGGCTGTGACATCAATGC-3′, 5′-GGGCTATGTCCAATTCCATTC-3′), and CXC motif chemokine ligand (CXCL) 8 (i.e., IL-8; 5′-TCCTGATTTCTGCAGCTCTGTG-3′, 5′-GGTCCACTCTCAATCACTCTC-3′). Target gene expression levels were normalized to β2-microglobulin (5′-TGTGCTCGCGCTACTC-TCTCTT-3′, 5′-CGGATGGATGAAACC-CAGACA-3′). Graphical representation of qPCR data was performed using GraphPad Prism 5.02.

### 2.7. Phagocytosis Assay

#### 2.7.1. Labelling of Bacteria

For the analysis of phagocytosis, Top10 *E. coli* were labeled before opsonization. Therefore, bacteria were adjusted to a concentration of 100 mg/mL in phosphate-buffered saline (PBS) and labeled with 0.36 μM eFlour 670 cell proliferation dye (Invitrogen) at 37 °C for 1 h. Following three washing steps with PBS/10% FCS, fixation was performed by incubating the bacteria in methanol for 1 min. Afterwards, the labeled bacteria were washed, aliquoted, dried (Concentrator plus; Eppendorf, Hamburg, Germany), and stored at −80 °C until use. Opsonization was performed as described above.

#### 2.7.2. Phagocytosis Assay

Differentiated THP-1 as well as MARCKS WT and KO cells (0.5–1 × 10^5^ cells/well in 100 μL standard medium, 96-well plate) were challenged at 37 °C with opsonized eFlour 670-labeled bacteria (>5 bacteria per cell in 100 μL standard medium). Concomitantly, negative control cells were kept on ice. After 60 min, phagocytosis was stopped by centrifugation at 4 °C and subsequent addition of stop solution (PBS, 2 mM EDTA, pH 8, 0.02% sodium azide). The phagocytic capacity was then assessed by flow cytometry. To discriminate living from dead cells, DAPI (final concentration: 1 μM; Biolegend) was added before measurement. Data were analyzed using FlowJo and GraphPad PRISM 5.02.

## 3. Results

### 3.1. Generation and Characterization of Monocytic MARCKS KO Cell Lines

**Figure 1:**
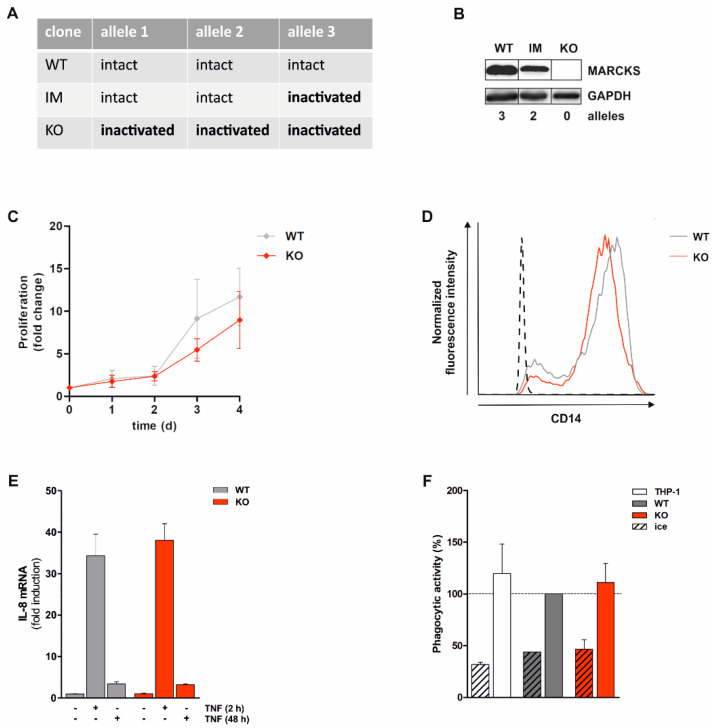
Generation and characterization of MARCKS KO cells. Monocytic THP-1 cells were modified using the CRISPR/Cas9 technique to generate MARCKS KO cells. The resulting WT, IM, and KO clones were characterized in terms of allelic integrity, MARCKS protein expression, proliferation, monocytic differentiation, cytokine expression, and phagocytosis. (**A**) The table summarizes the condition of the 3 MARCKS alleles in MARCKS WT, IM, and KO clones as determined by Sanger sequencing. (**B**) MARCKS protein levels were detected in whole cell extracts of MARCKS WT, IM, and KO clones. Loading control: GAPDH (representative experiment, n > 3). (**C**) MARCKS WT and KO cells were synchronized for 3 d in minimal growth medium. Afterwards, cells were transferred into standard growth medium and cell numbers were counted daily (mean ± SD, n = 3). (**D**) Following calcitriol-induced differentiation (100 nM) for 3 d, CD14 expression on the cell surface was assessed using flow cytometry (dashed line: isotype control; representative experiment, n > 10). (**E**) Five-day calcitriol-differentiated MARCKS WT and KO cells were incubated with 80 ng/mL TNF for 2 h or 48 h (TNF added at day 3), and the IL-8 mRNA level was determined by qRT-PCR. The IL-8 level in differentiated control cells (i.e., in the absence of TNF) was set as 1 (mean ± SD, representative experiment determined in triplicates; n = 3). (**F**) Five-day differentiated THP-1 as well as MARCKS WT and KO cells were incubated with opsonized eFlour670-labeled bacteria for 1 h (>5 bacteria per cell; mean ± SD, representative experiment, determined in duplicates; n = 4). During the incubation with bacteria, control cells were kept on ice. Phagocytosis was analyzed using flow cytometry. The phagocytic activity (i.e., the geometric mean fluorescence intensity) of living and bacteria-positive cells was calculated, and the value obtained in differentiated MARCKS WT cells was set as 100% (dashed line).

The functions of MARCKS in monocytic cells are only partially understood [23,24,25]. Therefore, THP-1 monocytic cells were modified using the CRISPR/Cas9 technique to generate MARCKS KO cells [27,28]. In THP-1 cells, three MARCKS alleles are present due to leukemia-associated abnormality [31]. Initially, we created KO cell lines in which all three alleles are inactivated as determined by Sanger sequencing (Figure 1A). In addition, we created cell lines exhibiting an intermediate (IM) phenotype in which two alleles remained intact, while one allele was inactivated. As controls, we used either WT clones derived from the CISPR/Cas9 procedure (i.e., clones in which the technique was not successful) or WT THP-1 cells. The protein level of MARCKS present in WT clones was partially reduced in IM cells and completely abolished in KO clones (Figure 1B). Cell proliferation was only weakly (not statistically significant) affected in KO cell lines (Figure 1C). Similarly, the cell differentiation was comparable in MARCKS KO cell lines compared to the WT controls (as monitored by CD14 expression; Figure 1D). Differentiated MARCKS WT and KO cells incubated with TNF for 2 h or 48 h showed a comparable expression of IL-8 (Figure 1E). Exposure to opsonized bacteria induced an increase in phagocytic activity, which was also not affected by MARCKS deficiency (Figure 1F). These data demonstrate that the removal of MARCKS does not affect cell proliferation, differentiation, IL-8 expression, and phagocytosis under the conditions applied.

### 3.2. PMA-Induced Total ROS Production Is Completely Abolished in Monocytic MARCKS KO Cells

**Figure 2:**
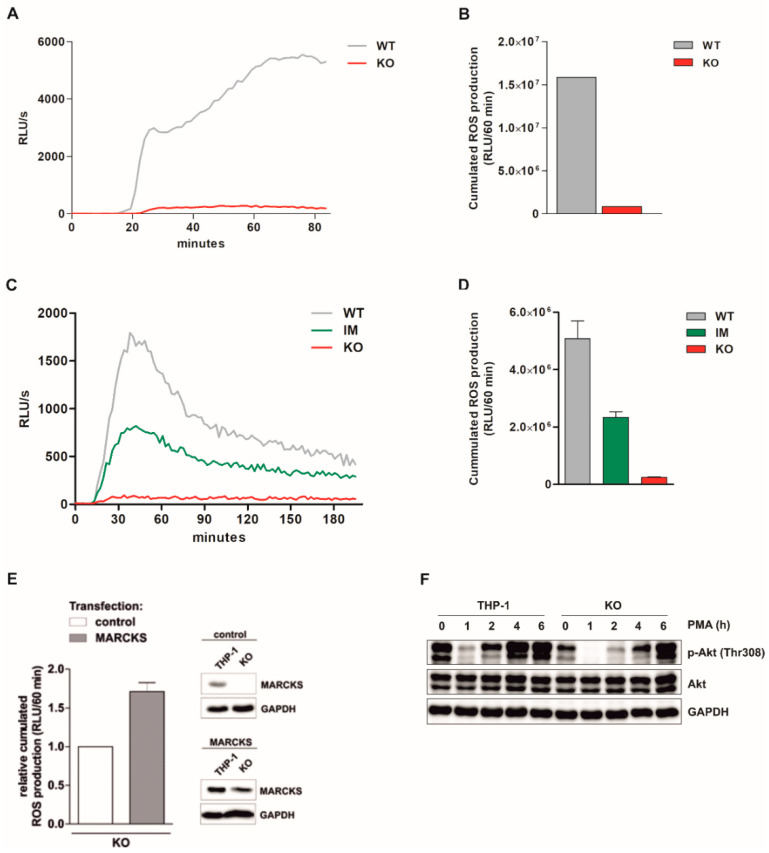
PMA-induced ROS production is suppressed in monocytic MARCKS KO cells. (**A**–**D**) Monocytic THP-1-derived MARCKS WT and KO clones were differentiated with 100 nM calcitriol for 5 d. ROS production was induced by 100 nM PMA and assessed via luminol-amplified chemiluminescence in relative light units per second (RLU/s). (**A**,**B**) PMA-induced total ROS production is completely abolished in monocytic MARCKS KO cells. Kinetics (**A**) and the respective cumulated total ROS production (i.e., the area under the curve (AUC) within 60 min following PMA stimulation) (**B**) of total ROS production in MARCKS WT and KO clones (representative experiment, n = 3). (**C**,**D**) PMA-induced intracellular ROS production is strongly inhibited in MARCKS KO and partially reduced in MARCKS IM cells. Kinetics ((**C**), representative experiment) and the respective cumulated intracellular ROS production within 60 min following PMA stimulation ((**D**), mean ± SD, n = 3) in WT, IM, and KO clones. (**E**) Reconstitution of MARCKS in KO cells restores PMA-induced ROS production. MARCKS KO clones were differentiated with 100 nM calcitriol for 5 d. At day 3, cells were transfected with a MARCKS-expressing vector or the respective vector control (control cells: THP-1, transfection control: EGFP-encoding plasmid). Cumulated ROS production of EGFP-positive (i.e., transfected) cells within 60 min following PMA stimulation was detected by flow cytometry. The ROS production in control-transfected KO cells was set as 1 (mean ± SD, n = 3). The insets show the MARCKS levels in KO and THP-1 cells transfected with the control or the MARCKS expression vector. (**F**) Basal Akt levels are reduced and Akt re-phosphorylation is delayed in MARCKS KO cells. THP-1 WT and MARCKS KO cells were treated with 100 nM PMA. At the indicated time points, cells were harvested, and total cell extracts were prepared. Akt phosphorylation (at Thr308) and total Akt levels were determined via Western Blot (loading control: GAPDH; representative experiment, n = 3).

Monitoring several independent monocytic functions in the absence of MARCKS (see also above), we identified an important monocytic function which is dramatically impaired in KO cells. THP-1-derived MARCKS WT and KO clones were differentiated with 100 nM calcitriol for 5 d. Total ROS production was induced by 100 nM PMA and assessed via a luminol-amplified chemiluminescence assay in relative light units per second (RLU/s). The kinetics of PMA-induced total ROS production in MARCKS WT and KO clones is shown in Figure 2A. The dramatic PMA-induced total ROS production in WT MARCKS clones was completely abolished in KO cells. The relative cumulated ROS production (i.e., the area under the curve, AUC) within 60 min following PMA stimulation for the experiment shown in A is depicted in Figure 2B. These experiments show that the MARCKS protein is absolutely indispensable for PMA-induced ROS production.

### 3.3. PMA-Induced Intracellular ROS Production Is Strongly Inhibited in MARCKS KO Clones and Reduced in MARCKS IM Cells

To further characterize the quality of ROS production, we particularly measured intracellular ROS production. For these experiments, we additionally used the IM clones (see Figure 1A,B). MARCKS WT, IM, and KO clones were differentiated with calcitriol, and intracellular ROS production was induced by PMA. The kinetics of intracellular ROS production in WT, IM, and KO clones assessed via chemiluminescence assay is shown in Figure 2C. For the same experiment, cumulated ROS production in WT, IM, and KO clones within 60 min is shown in Figure 2D. The data demonstrate that MARCKS is also essential for intracellular ROS production in monocytic cells. We also show that in IM clones only a partial reduction of ROS levels was observed, which suggests dose dependency.

### 3.4. Reconstitution of MARCKS in KO Cells Restores PMA-Induced ROS Production

Next, we tested whether ROS generation can be restored by re-transfection of exogenous MARCKS in MARCKS KO cells. Again, MARCKS KO clones were differentiated with calcitriol for 5 d. At day 3, cells were transfected with a MARCKS-expressing vector or the respective vector control. ROS production of EGFP-positive (i.e., transfected) cells was detected by flow cytometry and depicted as relative cumulated ROS production within 60 min following PMA stimulation. Transfection of KO cells with the MARCKS expression vector restored the MARCKS protein levels (Figure 2E, insets). In KO cells transfected with the MARCKS expression plasmid, a modestly increased ROS production was measured, compared to control cells (Figure 2E). These data show a direct dependence of ROS generation on the presence of MARCKS protein.

### 3.5. Basal Akt Levels Are Reduced and Akt Re-Phosphorylation Is Delayed in MARCKS KO Cells

The kinase Akt has been demonstrated to be involved in signaling leading to ROS formation [38]. To test whether this signaling is also affected by MARCKS deficiency, Akt phosphorylation in MARCKS KO cells was assessed. THP-1, MARCKS WT, and MARCKS KO cells were treated with PMA, and at the indicated time points, total cell extracts were prepared. Akt phosphorylation (Thr308) and total Akt levels were determined via Western Blot. In MARCKS KO cells, a reduced phosphorylation of Akt was found, compared to THP-1 and MARCKS WT cells (Figure 2F, data not shown). As described earlier, PMA exposure led to a decrease in Akt phosphorylation with a recovery at later time points. Interestingly, in MARCKS KO cells, this recovery of phosphorylation was retarded (Figure 2F). The experiments suggest that MARCKS is required for optimal Akt signaling.

### 3.6. Total ROS Production Is Markedly Reduced and Intracellular ROS Production Is Abolished in Bacteria-Exposed MARCKS KO Cells

**Figure 3:**
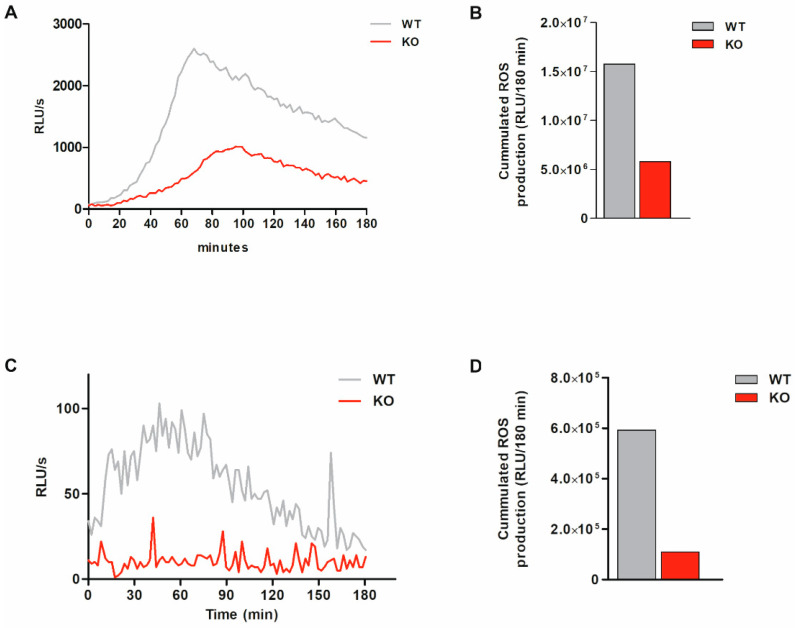
Bacteria-induced total ROS production is markedly inhibited, and intracellular ROS production is completely abolished in MARCKS KO cells. MARCKS WT and KO cells were differentiated with 100 nM calcitriol for 5 d. ROS production induced by opsonized bacteria (bac; >5 bacteria/cell) was assessed via luminol-amplified chemiluminescence in RLU/s. (**A**,**B**) Kinetics (**A**) and the respective cumulated total ROS production within 180 min (**B**) in MARCKS WT and KO clones following stimulation (representative experiment, n = 4). (**C**,**D**) Kinetics (**C**) and the respective cumulated intracellular ROS production within 180 min (**D**) in WT and KO clones following stimulation (representative experiment, n = 3).

As a second, (patho-)physiologically more relevant stimulus, and to avoid potential interfering effects due to PKC-dependent MARCKS phosphorylation in response to PMA in the WT [39], exposure to opsonized bacteria was applied to induce ROS production. Again, monocytic MARCKS WT and KO clones were differentiated with calcitriol and then exposed to bacteria for the indicated time points. Total and intracellular ROS production was assessed via chemiluminescence assay. In MARCKS KO cells exposed to bacteria, total ROS production is markedly reduced (Figure 3A,B), and the intracellular ROS generation is abolished (Figure 3C,D). Our results suggest that bacteria-induced total ROS production is strongly mediated by MARCKS, but not completely dependent on this protein, whereas bacteria-induced intracellular ROS formation completely depends on MARCKS.

### 3.7. ROS Production Is Suppressed by PKCβ KO and PKC Inhibitor Staurosporine

**Figure 4:**
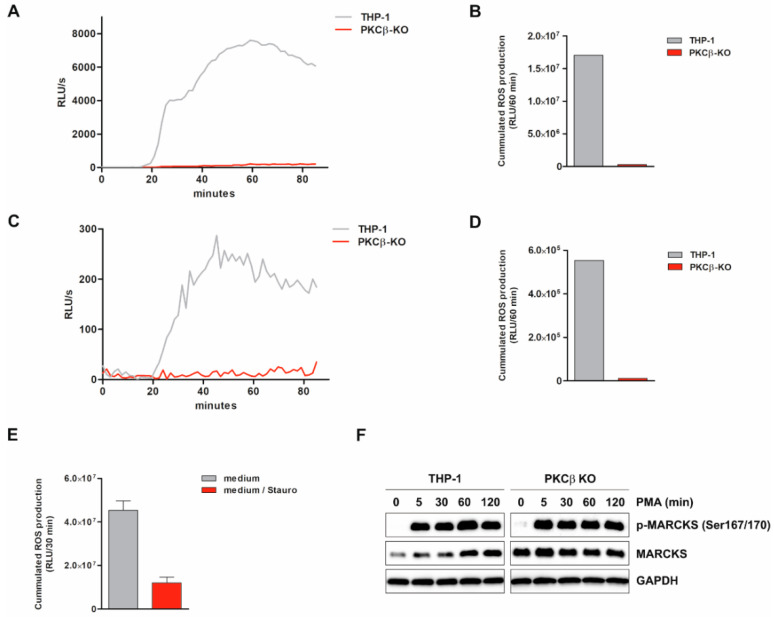
ROS production is suppressed by PKCβ KO and PKC inhibitor Staurosporine, and MARCKS level and Ser167/170 phosphorylation are not affected in PKCβ KO cells. THP-1-derived PKCβ KO cells (generated using the CRISPR/Cas9 technique) and THP-1 WT cells were differentiated with 100 nM calcitriol for 5 d. Subsequently, ROS production was induced by PMA (100 nM) and determined (in RLU/s) via luminol-amplified chemiluminescence. (**A**,**B**) Kinetics (**A**) and the respective cumulated production (i.e., the AUC) within 60 min following stimulation (**B**) of total ROS generated by differentiated THP-1 WT and PKCβ KO cells (representative experiment, n = 3). (**C**,**D**) Kinetics (**C**) and the respective cumulated production within 60 min following stimulation (**D**) of intracellular ROS generated by differentiated THP-1 and PKCβ KO cells (representative experiment, n = 3). (**E**) Primary human monocytes were incubated in medium with Staurosprine (Stauro; 80 nM) for 2.5 h. Subsequently, PMA-induced total ROS production was assessed and depicted as cumulated ROS production within 30 min following stimulation (mean ± SD; n = 2). (**F**) THP-1 WT and PKCβ KO cells were treated with 100 nM PMA. At the indicated time points, cells were harvested. Total cell extracts were prepared, and MARCKS ED phosphorylation (Ser167/170) and protein levels were determined (Western Blot; loading control: GAPDH; representative experiment, n = 3).

It has been shown that PKC is involved in signal transduction mediating ROS production [40]. Therefore, we examined if PKCβ is involved in ROS production under our condition. THP-1-derived PKCβ KO cells (generated using the CRISPR/Cas9 technique) and THP-1 WT cells were differentiated, and ROS production was induced by PMA and determined via luminol-amplified chemiluminescence. Neither total nor intracellular PMA-induced ROS production was detected in PKCβ KO cells (Figure 4A–D). Similar results were observed when opsonized bacteria were used as stimulus (data not shown). In addition, primary human monocytes were incubated in medium or with the PKC inhibitor Staurosporine (Stauro) for 2.5 h, and PMA-induced total ROS production was assessed. The presence of Stauro strongly inhibited the production of ROS under these conditions (Figure 4E). To examine whether PKCβ KO-associated effects on ROS production are mediated by an impairment of MARCKS, its level and ED phosphorylation at Ser167/170 were determined in PMA-treated WT and PKCβ KO cells. Our results indicate that PKCβ deficiency does not affect MARCKS expression or ED phosphorylation under the conditions tested (Figure 4F). The observation that the PMA- and bacteria-induced ROS production in monocytic cells is completely blocked in PKCβ KO cells and inhibited by PKC inhibition (similarly as observed in the absence of MARCKS) implicates that both molecules intimately work together under our conditions in monocytic cells to enable the production of ROS.

### 3.8. Monocytic ROS Production Is Increased by TNF Tong-Term Preincubation Which Is Dependent on MARCKS and PKCβ

**Figure 5:**
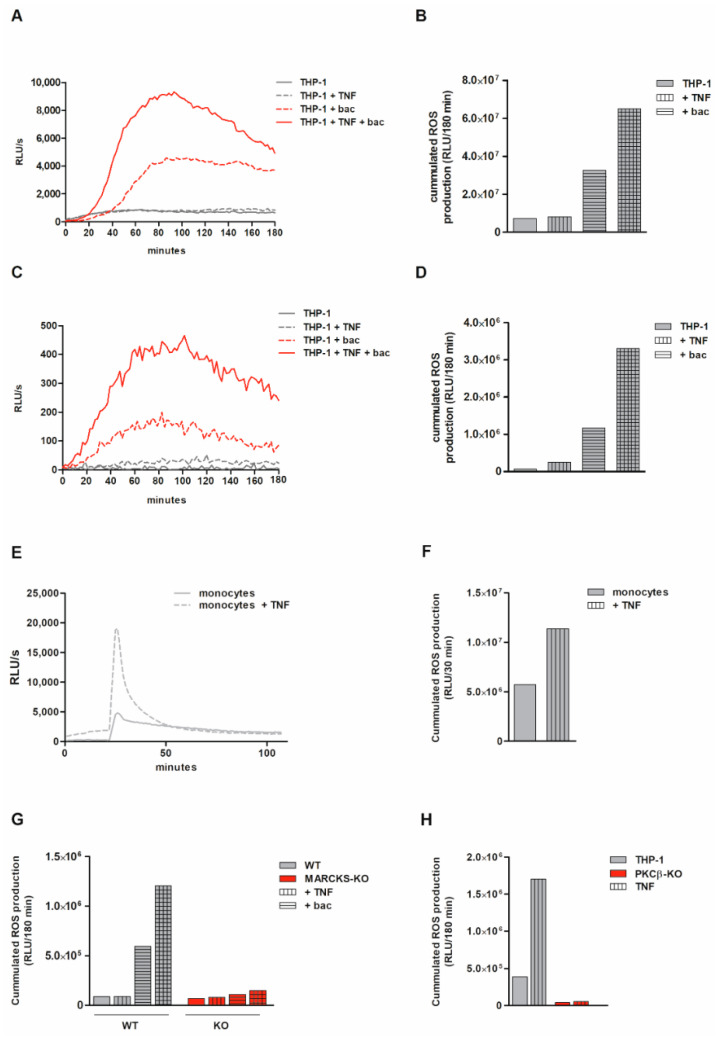
Increased monocytic ROS production following TNF long-term preincubation is dependent on MARCKS and PKCβ. (**A**,**B**) Kinetics (**A**) and the respective cumulated production (i.e., the AUC) within 180 min (**B**) of opsonized bacteria-induced (≥5 bacteria/cell) total ROS generated by 5 d calcitriol-differentiated THP-1 cells following preincubation with 80 ng/mL TNF for 48 h (i.e., added at day 3). Total ROS production was assessed via luminol-amplified chemiluminescence (in RLU/s) (representative experiment, n = 3). (**C**,**D**) Kinetics (**C**) and the respective cumulated production within 180 min (**D**) of opsonized bacteria-induced intracellular ROS generated by differentiated THP-1 cells following TNF preincubation as described in (**A**) (representative experiment, n = 3). (**E**,**F**) Kinetics (**E**) and the respective cumulated production within 30 min (**F**) of PMA-induced (100 nM) total ROS generated by primary human monocytes following TNF preincubation (80 ng/mL TNF for 48 h; representative experiment, n = 4). (**G**) Cumulated intracellular ROS production in MARCKS WT and KO clones (incubated as in (**A**)) within 180 min following stimulation with bacteria (representative experiment, n = 3). (**H**) Cumulated intracellular ROS production in THP-1 and PKCβ KO cells (incubated as in (**A**)) within 180 min following stimulation with bacteria (representative experiment, n = 3).

We have previously shown that long-term exposure to TNF induces phenomena of tolerance [26,33,41] and changes the immunological quality of monocytic cells [42]. Therefore, we investigated whether TNF long-term exposure affects the ROS production under our condition. For these experiments, different types of monocytic cells were preincubated with 80 ng/mL TNF for 48 h. Afterwards, ROS production was induced by bacteria or PMA. In TNF long-term-exposed THP-1 cells, a significantly higher bacteria-induced total (Figure 5A,B) and intracellular (Figure 5C,D) ROS production was found. Similar effects were found when PMA was used as a stimulus (data not shown). A significantly higher ROS production was also measured in TNF-preincubated primary human monocytes (Figure 5E,F). In MARCKS and PKCβ KO cells, both the direct stimulus-dependent effect (as described above) and the TNF-mediated enhancement of ROS production were completely abolished (Figure 5G,H). Our data demonstrate that under conditions of TNF tolerance, increased levels of ROS are generated. Both the direct stimulus-induced and the further TNF-enhanced ROS generation are absolutely dependent on MARCKS and PKCβ.

## 4. Discussion

MARCKS, initially perceived as an ordinary cytoskeleton-associated structural protein [5], emerged to be a surprisingly versatile protein due to its ability to shuttle among the cell membrane and the cytosol and to interact with a variety of different interaction partners [1,2]. In this way, it contributes to diverse cellular functions, such as cytoskeletal stability and rearrangements, cellular adhesion and migration, vesicle transport together with endo- and exocytosis, signal transduction, and gene expression [1,2]. Thus, MARCKS exerts an influence on a plethora of physiological and pathophysiological processes, such as embryonic development, tissue regeneration, neuronal plasticity, and inflammation [2]. The role of MARCKS in monocytic cells, however, has not been adequately addressed yet. Therefore, the aim of the study was to elucidate the impact of MARCKS on specific cellular processes of monocytic cells.

Monocytic THP-1 cells were modified using the CRISPR/Cas9 technique [27], leading to the establishment of MARCKS WT, IM (intermediate phenotype), and KO cells. Remarkably, the absence of MARCKS dramatically impaired both total and intracellular ROS production. This effect was independent of the stimulus used (PMA or opsonized bacteria) and could be reversed by re-transfection of MARCKS. The well detectable, but reduced ROS production of IM cells (when compared to KO or WT cells, respectively) further suggested a dose-dependent effect. Our data obtained using opsonized bacteria as an alternative and immunologically more relevant stimulus for ROS generation not only further supported the importance of MARCKS for ROS production. This approach had the additional advantage of avoiding the direct activation of PKC, thus circumventing potential interfering effects in WT cells due to PMA-induced and PKC-dependent MARCKS phosphorylation [39]. Our results also suggest that bacteria-induced total ROS production is markedly mediated by MARCKS, but not completely dependent on this protein, whereas bacteria-induced intracellular ROS formation appears to be completely dependent on MARCKS. By these experiments, we demonstrate for the first time that MARCKS is an essential regulator of ROS production in the monocytic cell type. To our knowledge, this mechanism has also not been shown in other cell types. Interestingly, it has been reported that, vice versa, ROS alters the expression and phosphorylation of MARCKS in various cell types [43,44]. MARCKS has also been shown to act as a crucial mediator in hydrogen peroxide-induced elevation of endothelial permeability, a process involving an increase in H_2_O_2_ levels (physiologically induced, for instance, by adenosine diphosphate [45] or angiotension II [46]), PKCδ activation, MARCKS phosphorylation, its translocation to the cytosol, and subsequent cytoskeletal rearrangements [47]. Therefore, MARCKS and ROS appear to be connected by mutual regulatory mechanisms.

In addition, MARCKS KO cells have been characterized in terms of proliferation, differentiation, cytokine/chemokine expression, and phagocytosis. The results of our analyses revealed that, with the exception of ROS production, MARCKS deficiency did not significantly impair the basic monocytic cell functions analyzed. For instance, cell proliferation was only weakly (and not statistically significant) affected in KO cell lines. Similarly, cell differentiation was comparable in both MARCKS KO and WT cells—as reflected by CD14 expression [48]. In an earlier report using murine macrophages, MARCKS KO had no influence on macrophage morphology or intracellular actin distribution [49]. Furthermore, the impact of MARCKS on cytokine expression that has been described in neutrophils [22] could not be observed for IL-8 as well as IL-1α/β, IL-6, and CCL20 (data not shown) in monocytic cells in the present study. Moreover, phagocytosis was not affected in our experiments. Though phagocytosis is an immunologic function associated with cellular transport processes [50] that were assumed to be influenced by MARCKS [7], it has also previously been shown that MARCKS deficiency had no influence on phagocytosis or micropinocytosis under most conditions [49]. Only for Zymosan-induced phagocytosis, for which a role of MARCKS was proposed earlier [21], was a mild reduction within a narrow time span described [49]. It is somewhat surprising that among several monocytic key functions, only ROS production as one specific function was identified to be so dramatically affected by the absence of MARCKS. It remains to be established whether MARCKS-like protein 1 (MARCKSL1, also known as MacMARCKS [51]), a MARCKS relative that shares a variety of its molecular characteristics [2], might play a compensatory role within other MARCKS-associated cellular processes.

Several data in the literature fit well to our present finding, demonstrating that MARCKS plays an essential role in ROS production. MARCKS is a potent sequestration agent for PI(4,5)P_2_ [6], and signaling mediated by PI(4,5)P_2_ and its derivatives plays central roles in ROS production [38] as well as PKC activation [52]. Furthermore, PKC is an important kinase for MARCKS [5], and PKC—including PKCβ [53,54]—has also been shown to exert important regulatory functions on ROS formation [40]. In our set of data, PMA- and bacteria-induced ROS production was completely blocked in PKCβ KO cells in a similar way as observed in MARCKS KO cells. Noteworthy, this effect was not mediated by an impairment of MARCKS in the PKCβ KO, since MARCKS levels and PMA-induced ED phosphorylation at Ser167/170 were not affected in these cells. Here, MARCKS ED phosphorylation may be mediated by alternative MARCKS-targeting kinases such as other PKC paralogues or ROCK [10]. With regard to ROS generation, this also implies that the remaining kinases are not able to compensate for the loss of PKCβ in the KO cells. This is somewhat surprising since PKCδ has especially been described to be involved in ROS generation in monocytes [55]. These differences may be due to the application of different stimuli (such as opsonized Zymosan) and the application of different experimental conditions (e.g., use of inhibitors). An equivalent inhibition of ROS production was found in primary human monocytes using the PKC inhibitor Staurosporine. This strongly suggests that both MARCKS and PKCβ are involved in systems of PMA- or bacteria-induced ROS production. Though MARCKS is a prominent target of PKCβ [56], it appears unlikely that MARCKS phosphorylation is a sufficient prerequisite for ROS formation under our conditions. Considering the unaffected (p-)MARCKS levels in PKCβ KO cells and the fact that PKC(β) can activate ROS generation via direct activation of the NAD(P)H oxidase complex [57,58], it is conceivable that MARCKS and PKCβ are both necessary for ROS formation but co-operate in a parallel fashion—though the involvement of a (potentially additional) vertically coordinated signaling cascade cannot be fully excluded. Our data also suggest that both molecules are not able to compensate for each other to a significant extent. Under our conditions, we also observed reduced levels of Akt phosphorylation and a retarded recovery of phosphorylation of this protein following PMA stimulation. Interestingly, PI(4,5)P_2_ has been shown to be involved in the activation of Akt [38]. On the other hand, both Akt and MARCKS (as mentioned above) are substrates of PKC.

We have found earlier that under conditions of TNF tolerance, the expression of proinflammatory cytokines and corresponding signaling pathways, such as the NF-κB system, are inhibited [42]. Here, we found in TNF long-term-exposed monocytic cells and primary monocytes that the formation of ROS is increased. The ROS system, therefore, appears to be one of the relatively few functionalities which are not inhibited but rather increased under monocytic TNF tolerance. Following short-term exposure to TNF, but not under tolerance conditions [33,41], it has previously been shown that ROS production is increased [59]. In MARCKS and PKCβ KO cells, the TNF-mediated enhancement of ROS production was completely abolished. Therefore, both the direct stimulus-induced ROS production (as already discussed above) and the TNF-induced further enhancement of ROS generation are absolutely dependent on MARCKS and PKCβ. Via the inhibition, but also activation, of specific cellular functions, tolerance is an important mechanism to resolve inflammation [42,60,61]. ROS is involved in the clearance of bacteria and viruses as well as the removal of proinflammatory products causing sterile inflammation [62,63]. Therefore, increased ROS under TNF tolerance may contribute to the termination of the inflammatory process.

## 5. Conclusions

In summary, the present study demonstrates that MARCKS is an essential molecule enabling ROS production by monocytic cells. Our data suggest that the MARCKS protein represents a thus far unknown piece in signaling networks responsible for ROS production.

## Data Availability

The data presented in this study are available upon request from the corresponding author.
